# Mechanical complications of hip spacers: a systematic review of the literature

**DOI:** 10.1007/s00402-022-04427-z

**Published:** 2022-04-12

**Authors:** Andrea Sambri, Michele Fiore, Claudia Rondinella, Lorenzo Morante, Azzurra Paolucci, Claudio Giannini, Calogero Alfonso, Massimiliano De Paolis

**Affiliations:** 1 IRCCS Azienda Ospedaliera Universitaria di Bologna, Via Massarenti 9, 40138 Bologna, Italy; 2 University of Bologna, Bologna, Italy

**Keywords:** Hip, Spacer, Prosthetic-joint infection, Complication, Mechanical

## Abstract

**Introduction:**

Temporary spacers used in the staged revision of a hip prosthetic joint infection (PJI) have been associated with several mechanical complications with very variable reported general complications rates up to 73%. The aim of this systematic review was to assess the mechanical complications associated with hip antibiotic-loaded spacers when treating periprosthetic hip PJI.

**Methods:**

Through an electronic systematic search of PubMed, articles reporting mechanical complications of spacers used in the treatment of hip PJI were reviewed. Dislocations, spacer fracture, femoral fractures, and acetabular lysis rates were evaluated.

**Results:**

Forty studies were included. Standardized molded spacers had a significantly higher weighted mean of total mechanical complication rates (37.2%) when compared to standardized preformed spacers (13.8%, *p* = 0.039), while no significant difference was found between molded spacers and manually shaped spacers. Spacer dislocation was the most frequent complication. No significant difference in mechanical complication rate was found between spacers with and without any metallic component.

**Conclusions:**

Spacer placement in chronic PJI of the hip with bone and soft-tissue defects is challenging and bears a high risk of mechanical failures and progressive bone loss during the interim period. A careful patient selection for spacer implantation is mandatory.

## Introduction

A staged revision is the “gold standard” for treating chronic prosthetic joint infection (PJI). The first stage includes implant and cement removal and bone and soft-tissue debridement, implantation of a temporary antibiotic-loaded cement spacer, and concomitant microbe-specific antibiotic treatment. Reimplantation with a new definitive implant can be done after infection resolution [1].

The use of antibiotic-loaded cement spacers allows for the local delivery of large amounts of antibiotics which has been shown to improve infection-related outcomes compared to resection arthroplasty [2, 3] Additional functions of an antibiotic-laden spacer are to allow for stability, maintenance of length, and patient mobility while waiting for infection eradication [12, 14–18]. Potential benefits of spacers are also improved function and less pain during the interval, [7, 9–13] shorter second stage, thanks to reduced intra-articular fibrosis and retraction [7, 9, 10, 12, 13].

In the literature, static and articulating spacers have been described [4, 5]. Recently, Lunz et al. [6] proposed a new “hip spacer classification system” to simplify comparison between different spacer designs. This include four categories: Resection arthroplasty (Girdlestone hip), Static spacer (PMMA cement cap implantation, either femoral or femoral + acetabular), hemi-spacer (comparable to a fixed-head hemiarthroplasty without implantation of an acetabular cap), and articulating spacer (Comparable to a total hip arthroplasty, articulation within the spacer). Hemi spacers and articulating spacers are mobile spacers and can be further categorized as either commercially available pre-formed components, commercially available molds, or custom-made.

Different methods and surgical techniques have been described for the fabrication of articulating spacers, including manually shaped (custom-made), standardized molded, standardized preformed, and antibiotic-coated prosthesis [7, 8].

Benefits of preformed cement spacers, such as spacer G (Tecres SpA, Verona, Italy), include longer antibiotic elution, the ability to mechanically load the component which further enhances antibiotic elution, and their availability in a variety of sizes, allowing for a closer approximation of native joint anatomy which may restore joint biomechanics and improve interstage function [9]. Intraoperatively manually shaped spacers are diverse and often include the addition of a central endoskeleton using a Kirschner wire (K-wire), Steinmann pin, or commercially created stainless-steel construct in the hopes of improving mechanical properties [10]. Prosthesis of antibiotic-loaded acrylic cement (PROSTALAC—DePuy Orthopaedics) has also been reported [11–13] and consists of a constrained cemented acetabular component and a femoral component with a modular head with antibiotic-loaded cement surrounding a stainless-steel endoskeleton.

Nonetheless, temporary spacers have been associated with several mechanical complications with very variable reported general complications rates ranging up to 73% [2, 14–18]. These include spacer fracture [18–20], bone fracture and lysis induced by stress on adjacent bone [19, 20] and implant dislocation, with loss of all benefit [19, 20].

Patients who require surgical intervention for mechanical complications of their spacer have lower infection cure rates and a worse final clinical hip evaluation compared to patients without any mechanical complications [19]. Thus, minimizing mechanical complications while treating infected prostheses is essential to optimize patient outcomes.

Recently, it has been shown that collective mortality following a two-stage protocol was underestimated [20]. Mechanical complications with the enclosed spacer can contribute to overall mortality, as there can be far-reaching consequences (further surgery with high multimorbidity, becoming bedridden, extended duration of treatment, and impaired functional outcome). Therefore, in hip two-stage revision for PJI, there is the question of whether to use an antibiotic-loaded cement spacer in the interval between stages. Several prior studies have been published looking at spacer failures and mechanical complication rates; however, the implant groups have been very heterogeneous.

The aim of this systematic review was to assess the mechanical complications associated with hip antibiotic-loaded spacers when treating periprosthetic hip PJI.

## Methods

This systematic review was conducted in accordance with the PRISMA (Preferred reporting items of systematic reviews) guidelines [21, 22].

The criteria used to select articles allowed to extrapolate data about the use of a cement spacer after hip prosthesis removal for a PJI. Studies eligible for this systematic review were identified through an electronic systematic search of PubMed and Web of Science, until 31st December 2021. The search string used was as follows: (hip) AND (spacer OR infection) AND (complication OR dislocation OR fracture OR rupture OR osteolysis).

Articles were included if published on a peer-reviewed journal. All duplicates were removed. Articles without an abstract were excluded from the study. Screening of the articles was done considering the relevance of titles and abstracts and looking for the full-text article when the abstract provided insufficient information about inclusion and exclusion criteria. Animal model studies, biomechanical reports, technical notes, letters to editors, cadaver or in vitro investigations, and instructional were excluded.

Articles that were considered relevant by electronic search were retrieved in full text and a hand-search of their bibliography was performed to find further related articles. Reviews and meta-analysis were also analyzed to broaden the search for studies. Articles with insufficient details about study populations, surgical intervention and type of reconstruction were excluded. Remnant studies were categorized by study type, according to the Oxford Centre for Evidence-Based Medicine.

All the included studies were reviewed, and data related to topics of interest were extracted and summarized (Tables 1, 2). The bias analysis according to Institute of Health Economics (IHE) quality appraisal checklist is performed Table 3).

**Table 1:** Characteristics of the spacers and their mechanical complications reported by the studies included in this review

Study	Year	Spacer design	Number of spacers	Mean age (year)	Metallic core	Mean spacer duration (months, approximated)	Mechanical complications, *n*. (%)	Spacer fracture, *n*. (%)	Perispacer fracture, *n*. (%)	Spacer dislocation, *n*. (%)	Acetabular complications, *n*. (%)
Buller et al.[22]	2021	Standardized—preformed	28	61.6	None	3.2	0	0	0	0	0
Burastero et al. [23]	2017	Standardized—preformed	71	67.4	Steel reinforcement rod	NA	7 (9.9%)	1 (1.4%)	1 (1.4%)	5 (7.0%)	0
Cabrita et al.[2]	2007	Manually shaped	37	54.6	None	NA	6 (16.2%)	1 (2.7%)	0	3 (8.1%)	2 (5.4%)
Cai et al. [24]	2021	Manually shaped	38	67.5	K-wires or femoral stem	24 temporary (N/A) 14 indefinite	4 (10.5%)	0	2 (5.3%)	2 (5.3%)	0
Erivan et al.[13]	2017	Manually shaped	26	71	K-wires and/or Steinmann pins / None	3	24 (92.3%)	5 (19.2%)	5 (19.2%)	11 (42.3%)	3 (11.5%)
Hsieh et al.[25]	2004	Manually shaped	58	59	K-wires	3.1	3 (5.2%)	2 (3.4%)	0	1 (1.7%)	0
Jung et al.[16]	2009	Standardized—moulded	88	70	None	3	36 (40.9%)	9 (10.2%)	12 (13.6%)	15 (17.0%)	0
Koo et al. [26]	2001	Standardized—moulded	22	56	None	1.5–4	1 (4.5%)	0	1 (4.5%)	0	0
Lancaster et al. [27]	2020	Standardized—preformed	58	59.8	Femoral stem	NA	11 (19.0%)	5 (8.6%)	2 (3.4%)	4 (6.9%)	0
Leuning et al.[15]	1998	Manually shaped	12	60	Plates. screws	4	7 (58.3%)	1 (8.3%)	0	5 (41.7%)	1 (8.3%)
Magnan et al.[17]	2001	Standardized—preformed	10	71.8	Steel reinforcement rod	5	3 (30.0%)	0	0	1 (10.0%)	2 (20.0%)
Molinas et al.[28]	2017	Standardized—preformed	71	70	Steel reinforcement rod	NA	8 (11.3%)	0	0	8 (11.3%)	0
Petis et al.[29]	2017	Manually shaped	17	78.5	None	> 24	6 (35.3%)	0	1 (5.9%)	1 (5.9%)	4 (23.5%)
Pizzo et al.[30]	2020	Manually shaped	15	64.5	None	3.3	0	0	0	0	0
Shin et al. [31]	2002	Manually shaped	8	N/A	Femoral stem	NA	0	0	0	0	0
Yang et al.[32]	2019	Standardized—moulded	31	56	K-wires	3.3	14 (45.2%)	3 (9.7%)	4 (12.9%)	7 (22.6%)	0
Zhang et al.[33]	2020	Manually shaped	13	59.7	K-wires	5.2	12 (92.3%)	5 (38.5%)	1 (7.7%)	3 (23.1%)	3 (23.1%)
			10	61.1	Femoral stem	4.2	6 (60.0%)	0	0	3 (30.0%)	3 (30.0%)
			13	64.7	ACP + polyethylene sockets	9	2 (15.4%)	0	1 (7.7%)	1 (7.7%)	1 (7.7%)
Abendschein et al.[34]	1992	Manually shaped	1	N/A	None	1.1	0	0	0	0	0
Takahira et al.[35]	2003	Manually shaped	8	67.1	Ender nail/K-wires with cerclage wires	1.5–4.4	0	0	0	0	0
Fink et al.[36]	2008	Manually shaped	36	69	Femoral stem	1.5	1 (2.8%)	0	0	1 (2.8%)	0
Pattyn et al.[38]	2011	Standardized—preformed	61	65.4	Steel reinforcement rod	1.7	11 (18.0%)	0	1 (1.6%)	10 (16.4%)	0
D'angelo et al. [38]	2011	Standardized—preformed	28	71.4	Steel reinforcement rod	1.3	3 (10.7%)	0	0	3 (10.7%)	0
Romanò et al.[8]	2012	Standardized—preformed	183	60.3	Steel reinforcement rod	4	35 (19.1%)	0	5 (2.7%)	30 (16.4%)	0
Jones et al.[6]	2019	Standardized—moulded	30	64	Steel reinforcement rod	3.2	23 (76.7%)	7 (23.3%)	12 (40.0%)	4 (13.3%)	0
		Manually shaped	97		Various steel reinforcement rods		6 (6.2%)	2 (2.1%)	1 (1.0%)	3 (3.1%)	0
			56		ACP + polyethylene sockets		11 (19.6%)	0	5 (8.9%)	6 (10.7%)	0
		Standardized—preformed	2		Steel reinforcement rod		0	0	0	0	0
Bori et al. [18]	2013	Standardized—preformed	74	72.4	Steel reinforcement rod	1.2	8 (10.8%)	0	0	8 (10.8%)	0
Duncan et al.[39]	1993	Standardized—preformed	15	N/A	Steel reinforcement rod	1–9.2	3 (20.0%)	0	0	3 (20.0%)	0
Ivarsson et al. [40]	1994	Manually shaped	5	69.4	None	0.7–2	2 (40.0%)	0	1 (20.0%)	1 (20.0%)	0
Younger et al. [10]	1997	Standardized—preformed	61	67	Steel reinforcement rod	1.2–9.7	6 (9.8%)	0	1 (1.6%)	5 (8.2%)	0
Durbhakula et al. [41]	2004	Standardized—moulded	20	69.8	Rush pin	2.3–4.8	4 (20.0%)	2 (10.0%)	0	2 (10.0%)	0
Barrack et al.[9]	2002	Manually shaped	12	N/A	Rush pin	1.5–4	0	0	0	0	0
Deshmukh et al. [42]	1998	Manually shaped	5	N/A	Küntcher nail	NA	0	0	0	0	0
McGrory et al. [43]	2002	Manually shaped	1	N/A	Endoprosthetic head	NA	0	0	0	0	0
Isiklar et al.[44]	1999	Manually shaped	10	N/A	Steinmann pins	0.7–3.2	2 (20.0%)	1 (10.0%)	0	1 (10.0%)	0
Jahoda et al.[45]	2003	Manually shaped	29	N/A	K-wires	1.5–6.4	7 (24.1%)	2 (6.9%)	0	5 (17.2%)	0
Kraay et al.[46]	1992	Manually shaped	7	N/A	Cerclage wire	NA	0	0	0	0	0
Morimoto et al. [47]	2003	Manually shaped	1	16	Gamma locking nail	1.5	0	0	0	0	0
Yamamoto et al. [48]	2003	Standardized—moulded	17	61.8	K-wires	4.3	2 (11.8%)	1 (5.9%)	0	1 (5.9%)	0
Zilkens et al.[49]	1990	Standardized—preformed	1	71	Metallic telescopic shaft	6	0	0	0	0	0
Wentworth et al. [50]	2002	Standardized—preformed	135	N/A	Steel reinforcement rod	NA	15 (11.1%)	0	0	15 (11.1%)	0
Faschingbauer et al. [14]	2015	Standardized—moulded	93	69.3	Stainmann pin / None	NA	13 (14.0%)	12 (8.7%)	2 (1.4%)	12 (8.7%)	1 (0.7%)
		Manually shaped	45				14 (31.1%)				
Total	–	–	1659	–	–	–	316 (19.0%)	59 (3.5%)	58 (3.5%)	180 (10.8%)	20 (1.2%)

**Table 2:** Summarized data on the weighted means of the mechanical complications rates reported by the individual studies, according to the type of the spacer

Group	Number of spacers	Mechanical complications, % ± SD	Spacer fracture, % ± SD	Perispacer fracture, % ± SD	Spacer dislocation, % ± SD	Acetabular complications, % ± SD
Standardized preformed	798	13.8 ± 5.2	0.8 ± 2.2	1.3 ± 30.5	11.5 ± 4.3	0.3 ± 2.2
Standardized molded	301	37.2 ± 21.6	10.2 ± 6.3	13.5 ± 12	13.5 ± 6.9	0
Manually shaped	560	19.2 ± 24.7	3.7 ± 7.2	3.3 ± 5.2	9.1 ± 11.2	3.3 ± 6.9
*p value*	*–*	0.047*	0.005*	< 0.001*	*0.477*	*0.156*
Total	*1659*	*19* ± *16.3*	*3.5* ± *5.9*	*3.5* ± *6.6*	*10.8* ± *7.5*	*1.2* ± *4.5*

*SD* standard deviation

*Statistically significant

**Table 3:** Institute of Health Economics (IHE) quality appraisal checklist for case series included in this review

Study	Q1	Q2	Q3	Q4	Q5	Q6	Q7	Q8	Q9	Q10	Q11	Q12	Q13	Q14	Q15	Q16	Q17	Q18	Q19	Q20	Total (yes/no/unclear)
Buller et al.	1	0	0	1	1	0	0	1	0	1	0	1	1	1	1	1	0	1	1	1	13/7/0
Burastero et al.	1	0	0	0	1	1	1	1	0	1	0	1	1	1	1	0	0	1	1	1	13/7/0
Cabrita et al.	1	1	0	0	1	1	1	1	1	0	0	1	0	1	1	1	0	1	1	0	13/7/0
Cai et al.	1	0	0	1	1	1	1	1	0	1	0	1	0	1	1	1	0	1	1	1	14/6/0
Erivan et al.	1	0	0	0	1	1	N/A	1	1	1	0	1	0	1	1	1	0	1	1	1	13/6/1
Hsieh et al.	1	0	0	1	1	1	N/A	1	0	1	0	1	0	1	1	0	0	1	1	1	12/7/1
Jung et al.	1	0	0	0	0	1	N/A	1	1	1	0	1	0	0	1	0	0	1	1	1	10/9/1
Koo et al.	0	0	0	0	1	0	0	1	1	1	0	1	1	0	1	1	0	1	1	0	10/10/0
Lancaster et al.	1	0	0	0	1	0	N/A	1	0	1	0	1	1	1	1	1	0	1	1	1	12/7/1
Leuning et al.	0	0	0	0	1	0	0	1	1	1	0	1	1	1	1	0	0	1	1	0	10/10/0
Magnan et al.	0	0	0	0	1	0	0	1	1	1	0	1	1	0	1	0	0	1	1	1	10/10/0
Molinas et al.	1	0	0	0	1	0	1	1	0	1	0	1	1	1	1	0	0	1	1	1	12/8/0
Petis et al.	1	0	0	0	1	1	N/A	1	1	1	0	1	1	1	1	1	1	1	1	1	15/4/1
Pizzo et al.	1	0	0	1	1	1	N/A	1	1	0	0	1	0	0	1	1	0	1	1	1	12/7/1
Shin et al.	1	1	0	N/A	1	0	N/A	1	0	0	0	1	0	N/A	1	0	0	1	N/A	1	8/8/4
Yang et al.	1	1	0	N/A	1	1	1	1	1	1	0	1	0	1	1	1	1	1	1	1	16/3/1
Zhang et al.	1	0	N/A	0	1	1	1	1	1	1	0	1	1	1	1	1	1	1	1	1	16/3/1
Abendschein t al.	1	0	N/A	N/A	1	0	N/A	1	0	0	0	1	0	N/A	1	0	0	1	1	0	7/9/4
Takahira et al.	0	1	N/A	N/A	0	0	0	1	1	0	0	0	0	0	1	0	0	1	0	0	5/13/2
Fink et al.	1	1	N/A	1	1	0	0	1	1	1	0	1	1	0	1	1	0	1	1	1	14/5/1
Pattyn et al.	1	1	N/A	1	0	0	N/A	1	1	0	0	1	0	0	1	0	0	0	1	1	9/ 9/2
D'angelo et al.	1	1	0	N/A	0	0	0	1	1	0	0	1	1	1	1	0	1	1	1	1	12/7/1
Romanò et al.	1	0	0	1	0	0	N/A	1	1	0	0	1	1	0	1	1	1	1	1	1	12/7/1
Jones et al.	1	0	N/A	1	1	0	N/A	1	1	1	0	1	1	1	1	1	1	1	1	1	16/2/2
Bori et al.	1	0	0	0	1	0	0	1	1	1	0	1	1	1	1	0	1	1	1	1	13/7/0
Duncan et al.	1	0	0	1	1	N/A	N/A	1	1	0	0	1	0	N/A	1	1	N/A	0	0	N/A	8/7/5
Ivarsson et al.	0	0	0	0	1	0	N/A	1	1	0	0	1	0	0	0	0	0	1	1	0	6/13/1
Younger et al.	1	1	0	1	1	0	1	1	0	1	0	1	1	1	1	0	1	1	0	0	13/7/0
Durbhakula et al.	1	0	0	1	1	0	1	1	1	0	0	1	0	N/A	1	1	0	1	1	0	11/8/1
Barrack et al.	1	0	0	0	1	0	1	1	1	0	0	0	0	0	1	1	0	1	1	0	9/11/0
Desmukh et al.	1	1	0	0	0	0	1	1	1	0	0	0	0	0	1	0	0	1	1	1	9/11/0
McGrory et al.	1	1	0	0	1	0	1	1	1	0	0	0	0	0	1	0	0	1	1	0	9/11/0
Isiklar et al.	1	0	0	N/A	1	0	1	1	1	0	0	1	0	0	1	1	0	1	1	0	10/9/1
Jahoda et al.	1	0	0	1	1	0	1	1	0	0	0	1	0	1	1	N/A	N/A	1	1	0	10/8/2
Kelm et al.	1	0	0	1	1	0	0	1	0	0	0	1	0	0	0	0	0	0	1	0	6/14/0
Kraay et al.	1	1	0	0	0	0	0	1	1	0	N/A	0	0	0	1	N/A	0	1	1	0	7/11/2
Morimoto et al.	1	0	0	0	1	0	1	1	1	0	0	1	0	1	1	0	1	1	0	1	11/9/0
Yamamoto et al.	0	0	0	0	1	0	1	1	1	0	0	1	0	1	1	0	1	1	0	1	10/10/0
Zilkens et al.	1	0	0	0	1	1	0	1	1	1	0	0	N/A	0	0	1	0	1	1	0	9/10/1
Wentworth et al.	1	0	1	0	1	1	1	1	1	0	0	0	1	1	1	1	1	1	1	1	15/5/0
Faschingbauer et al.	1	0	0	0	0	0	1	1	1	0	0	0	0	1	0	0	1	1	1	1	9/11/0

Q1: was the hypothesis/aim/objective of the study clearly stated?

Q2: was the study conducted prospectively?

Q3: were the cases collected in more than one centre?

Q4: were patients recruited consecutively?

Q5: were the characteristics of the patients included in the study described?

Q6: were the eligibility criteria (i.e., inclusion and exclusion criteria) for entry into the study clearly stated?

Q7: did patients enter the study at a similar point in the disease?

Q8: was the intervention of interest clearly described?

Q9: were additional interventions (co-interventions) clearly described?

Q10: were relevant outcome measures established a priori?

Q11: were outcome assessors blinded to the intervention that patients received?

Q12: were the relevant outcomes measured using appropriate objective/subjective methods?

Q13: were the relevant outcome measures made before and after the intervention?

Q14: were the statistical tests used to assess the relevant outcomes appropriate?

Q15: was follow-up long enough for important events and outcomes to occur?

Q16: were losses to follow-up reported?

Q17: did the study provided estimates of random variability in the data analysis of relevant outcomes?

Q18: were the adverse events reported?

Q19: were the conclusions of the study supported by results?

Q20: were both competing interests and sources of support for the study reported?

Studies with reported quantitative data were used for statistical analysis. The weighted mean was calculated to summarize the complication rates reported in the individual studies and to compare them according to the type of spacer used. The Shapiro–Wilk test was used to verify normal distribution. The Levene test was used to assess the equality of variances. As parametric test, the two-tailed unpaired Student T test was used to compare the weighted mean (WM) values between two unpaired groups, in case of equality of the variances, otherwise the Welch T test was used. The one-way ANOVA test was used to compare more than two unpaired groups, using the Tukey HSD ("Honestly Significant Difference") post hoc test, to indicate which groups were significantly different from which others. Pearson’s coefficient was used to make correlations. Spearman rho was used to identify monotonic relationship between variables (age and mean spacer duration and mechanical complication rates). *P* value < 0.05 was considered to be significant. All statistical analyses were performed with IBM SPSS v26.0 for MacOS (SPSS Inc., Chicago, Illinois).

## Results

**Fig. 1:**
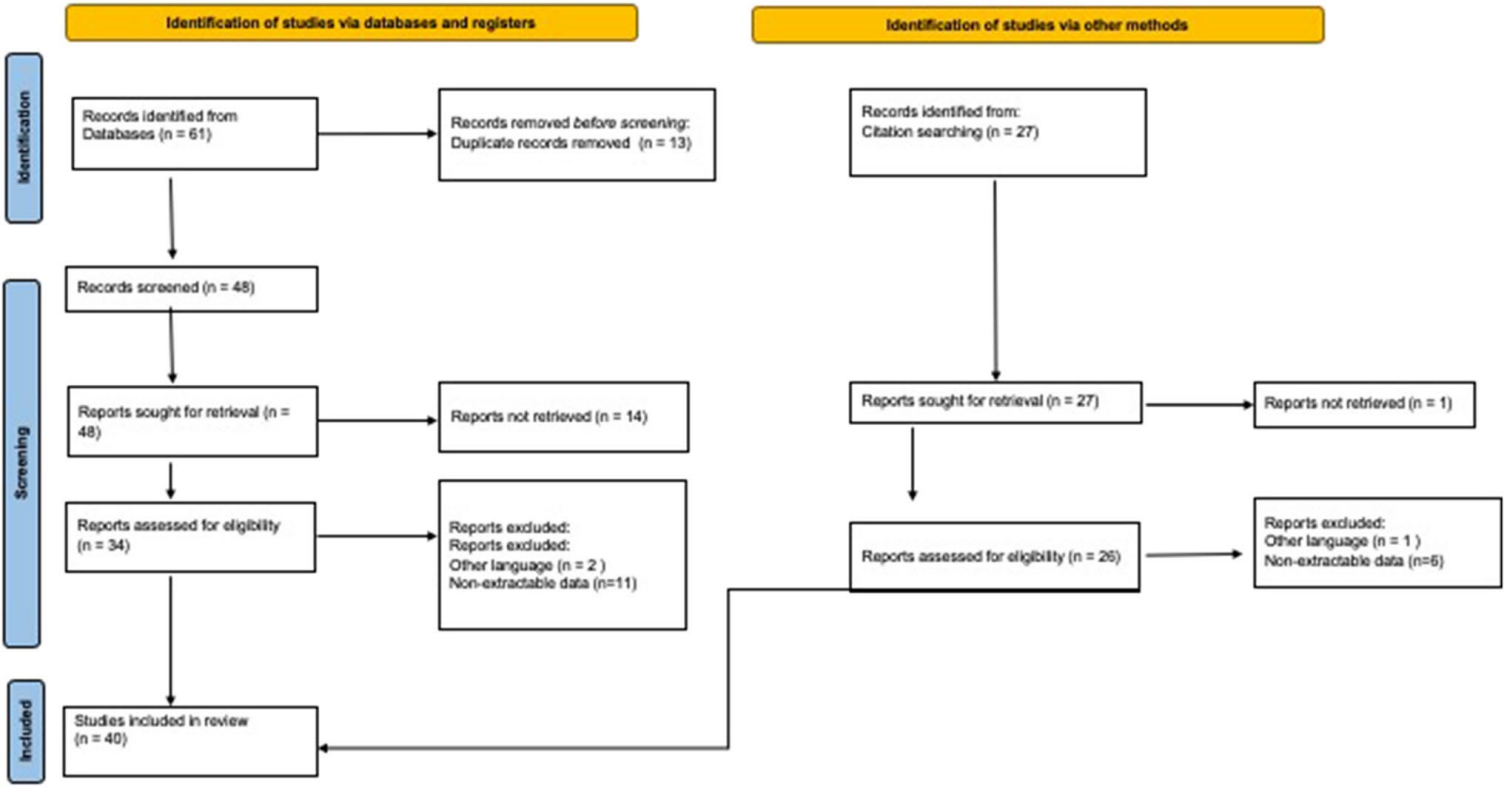
PRISMA flow diagram and the selection of studies

A total of 21 studies were found through the electronic search and 19 studies were added after cross-referenced research on the bibliography of the examined full-text articles. After a preliminary analysis, a total of 40 studies were included in this systematic review (Table 1, Fig. 1).

Thirteen studies reported the results of treatments in which a standardized prefabricated spacer was used [9, 11, 18, 19, 23–32], 5 studies reported on spacers that were intraoperatively produced by means of standardized molds or based on the method of Ivarson et al. [33] (by separately duplicating the shape of the retrieved femoral head and the femoral stem component with bone cement) [17, 31, 34–36], 20 studies reported on intraoperatively manually shaped spacers [2, 10, 14, 16, 33, 37–51], and two studies presented series in which different types of spacers were used. [7, 15] (Table 1).

Most of the spacers, regardless of the type, contained or were molded around a metallic core. The metallic component used was variable and included K-wires, Steinmann pins, rush pins, intramedullary nails, plates, steel rods, cerclages, femoral stems, and antibiotic-coated prostheses (Table 1). In detail, 30 studies reported on spacers with a metallic core [9–11, 16, 18, 19, 24–32, 35–38, 41, 42, 44–51], 8 studies on metal-free spacers [2, 17, 23, 33, 34, 39, 40, 43], and two studies presented a series in which spacers both with and without metal components were included [14, 15] (Table 1).

A total of 1659 spacers were included in this review from the studies that met the inclusion criteria. There were 798 standardized preformed spacers, 301 standardized molded spacers in 8 studies, and 560 manually shaped spacers in 22 studies (Tables 1, 2). A total of 1308 spacers included metallic components and 213 spacers resulted metal-free, while for 138 spacers, the presence of metal was not specified (Tables 1, 2).

In a pooled analysis, the overall mechanical complications rate ranged from 0% to 92.3% of the spacers implanted, with a weighted mean (WM) of 19% ± standard deviation (SD) = 16.3% and a total of 316 events. In detail, there were a total of 59 spacer fractures (WM: 3.5% ± SD = 5.9%, range: 0–38.5%), 58 peri-spacer femoral fractures (WM: 3.5% ± SD = 6.6%, range: 0–40%), 180 dislocations of the spacer (WM: 10.8% ± SD = 7.5%, range: 0–42.3%), and 20 acetabular complications, including acetabular lysis and pelvic protrusion (WM: 1.2% ± SD = 4.5%, range: 0–23.1%) (Table 1). Thirty-eight studies reported clearly recognizable data on mechanical complications according to the type of spacer used.

A significant difference was found comparing the mechanical complication rates among the 3 groups of different types of spacers (*p* = 0.047), and particularly comparing the incidence of spacer fractures (*p* = 0.005) and peri-spacer femoral fractures (*p* < 0.001) (Table 2). In detail, standardized molded spacers appeared to have a significantly higher weighted mean of total mechanical complication rates (WM: 37.2% ± SD = 21.6%) when compared to standardized preformed spacers (WM: 13.8% ± SD = 5.2%, *p* = 0.039), while no significant difference was found between molded spacers and manually shaped spacers, despite a strong trend of higher complication rates observed for molded spacers (Table 2). Moreover, a higher incidence of spacer fractures and peri-spacer femoral fractures was found when a standardized molded spacer was used (WM: 10.2% ± SD = 6.3% and WM: 13.5% ± SD = 12%, respectively), both compared with standardized preformed spacers (WM: 0.8% ± SD = 2.2%, *p* = 0.004 and WM: 1.3% ± SD = 30.5%, p < 0.001, respectively) and manually shaped spacers (WM: 3.7% ± SD = 7.2%, *p* = 0.039 and WM: 3.3% ± SD = 5.2%, *p* = 0.001, respectively) (Table 2). No significant difference was found comparing weighted means of complication rates between standardized preformed spacers and manually shaped spacers (Table 2). No significant difference was found on spacer dislocations and acetabular complications among the groups (Table 2).

The presence of a metallic core was recognizable in 39 studies. No significant difference in mechanical complication rate was found between spacers with and without any metallic component, (WM: 18.2% ± SD = 18.6% versus 23.2% ± SD = 17.6%, *p* = 0.477) and specifically regarding spacer fractures (WM: 2.8% ± SD = 6.1% versus 4.5% ± SD = 1.7%, *p* = 0.446), peri-spacer fractures (WM: 3.1% ± SD = 6.8% versus 6.8% ± SD = 6.5%, *p* = 0.155), spacer dislocations (WM: 11.3% ± SD = 7.8% versus 9.1% ± SD = 7.5%, *p* = 0.457), and acetabular complications (WM: 1% ± SD = 4.2% versus 2.7% ± SD = 6.3%, *p* = 0.348) (Table 4).

**Table 4:** Summarized data on the weighted means of the mechanical complications rates reported by the individual studies, according to the presence of a metallic core

Group *§*	Number of spacers	Mechanical complications, % ± SD	Spacer fracture, % ± SD	Perispacer fracture, % ± SD	Spacer dislocation, % ± SD	Acetabular complications, % ± SD
Metallic core	1308	18.2 ± 18.6	2.8 ± 6.1	3.1 ± 6.8	11.3 ± 7.8	1 ± 4.2
No metallic core	220	23.2 ± 17.6	4.5 ± 4.7	6.8 ± 6.5	9.1 ± 7.5	2.7 ± 6.3
*p value*	*–*	*0.477*	*0.446*	*0.155*	*0.457*	*0.348*

*SD* standard deviation

^§^Data available for 39 out of 40 studies

No correlation was found between patients’ age and mechanical complications rate. In 28 studies, it was possible to correlate the mean of the spacer persistence time and the rate of mechanical complications. No statistically significant correlation was found, although there was a trend toward increasing complications with increasing spacer persistence time (*p* = 0.124), particularly for hand-modeled spacers (*p* = 0.063).

## Discussion

Articulating hip spacers help to improve the joint function, enable early mobilization of the patient, and better preserve limb length and periarticular tissues. This leaves the surgical area more suitable for reimplantation, especially on the acetabular side [17, 52]. Different methods and surgical techniques have been described in the literature for the fabrication of articulating spacers, including handmade, molded intraoperatively, prefabricated, and antibiotic-coated prostheses [7, 8].

Many studies reported on mechanical complications rates in articulating hip spacers, with complications rates ranging between 0% and more than 50% [2, 9–11, 14–19, 23–51]. However, most of the series in the literature are relatively small and very heterogeneous. Moreover, only a few of them directly compared complications rates in different types of spacers [7, 15]. To the best of our knowledge, this is the first systematic review focusing on mechanical complications of hip spacers.

Among 40 studies analyzed, mechanical complications were found in 316 (19.0%) of the 1659 cases. Mechanical complications were more frequent in the case of a standardized molded spacer. A difference between handmade spacers and molded spacers was also reported by Jones et al. [7] and Anagnostakos et al. [53], even if the inhomogeneity of the patient population meant that this was not statistically significant. On the other hand, Faschingbauer et al. [15] reported a higher number of complications with manually shaped spacers, without differentiation on the type of complication.

Spacer dislocation is the most frequently reported complication (10.8%). However, no differences were observed among singular types of spacers. Widely divergent dislocation rates have been reported in the literature. Jung et al. [17] reported a dislocation rate of 17%, whereas Magnan et al. [18] in a small series of 10 cases reported a dislocation rate of 10% after implantation of a standardized hip spacer. Faschingbauer et al. [15] observed a dislocation rate of 8.7% which is clearly less than that reported by Leunig et al. (41.7%). [16] On the other hand, Buller et al. [21], Koo et al. [34], Shin et al. [41], and Takahira et al. [44] did not observe any dislocations following the implantation of standardized spacers.

A few series reported risk factors for spacer dislocation. Cabrita et al. [2] observed an increased risk of dislocation when the necks of these spacers were too valgus, which facilitated lateral migration. The dislocation rate tended to be higher with smaller off-set, an association that was statistically significant in the study by Leunig et al. [20]. It was also hypothesized an increased risk of spacer dislocation if the patient is non-compliant or cannot tolerate partial weight-bearing, if the size of the spacer is too small and if large osseous defects of the acetabulum do not allow for normal spacer articulation [7, 54]. Bori et al. [19] claimed that the rate of dislocation was also higher in patients with a prior dislocation of the prosthesis and lower in patients who underwent hip arthroplasty after arthritis compared to those who underwent hemiarthroplasty after proximal hip fractures. Moreover, an increased risk of spacer dislocation was reported in large bone defects in the acetabulum and in patients with muscular insufficiency because of lower off-set of the hip, limb length shortening, or absence of the abductor muscles. Nonetheless, Molinas et al. [26] reported that lateral and vertical femoral off-set did not modify dislocation rate.

To reduce the risk of dislocation, Burastero et al. [24] proposed the use of an acetabular custom-made spacer in addition to the femoral one. Pizzo et al. [40] reported on the use of constrained liners, which was shown to be highly successful in preventing prosthetic hip dislocation in patients at high risk for recurrent instability.

In the case of dislocation, Faschingbauer et al. [15] observed that closed reduction and stable retention were possible in only 4 out of 12 dislocations, whereas all other patients with a spacer dislocation underwent a subsequent operation with spacer revision. Because dislocation may recur, treatment with orthosis or skin traction has been encouraged.

Spacer fracture was reported in 3.5% of the 1659 cases reported in the literature. A higher rate of spacer fracture was observed in standardized molded spacers. In comparison to intraoperatively molded cement spacers, prefabricated cement spacers are manufactured to maximize strength.

Similar rates of dislocations were reported whichever approach was used for revision surgery [55], despite most of the series performed revision surgery with a direct lateral approach to the hip [9, 18].

In the series by Faschingbauer et al. [15], 50% of patients with a spacer fracture remained asymptomatic and showed a stable condition, while the other half underwent spacer revision.

To prevent a spacer fracture, Jung et al. [17] suggested the insertion of a metallic endoskeleton into the spacer. Jones et al. [7] reported that spacer fractures were only seen in molded or handmade spacers with no spacer fracture in the antibiotic-coated prosthesis group. Even though a higher rate of spacer fracture was observed in those with no metallic core (4.5% vs 2.8%), this was not statistically significant. However, it must be considered that metallic cores included in different series of the literature are very heterogeneous, ranging from K-wires and Steinmann pins to antibiotic-coated prosthesis.

Laboratory testing has shown improved strength with reinforced spacers; however, clinical evidence is lacking [15]. Schöllner et al. [56] investigated the mechanical properties of gentamicin-loaded hip spacers after the insertion of K-wires in vitro. The insertion of the K-wires prevented any dislocation of the spacer fragments, but did not significantly improve mechanical properties. The mechanical stability of spacers is determined and influenced by many parameters, including geometry, aging, storage, type of cement, the type and content of antibiotic, the presence of an endoskeleton, and standardization of its preparation (such as atmospheric composition during mixing and the frequency and duration of the particular mixing process) [16]. In addition to the manufacturing process, other factors might compromise the function of the spacer, including the residual bone quality after the first surgery, or deficient soft tissue.

Femoral peri-spacer fractures are a common finding when dealing with hip PJI, observed in 3.5% of the cases in the Literature, with a significantly higher incidence among standardized molded spacers. It should be noted that some predisposing factors may lead to a femoral fracture, including osteoporosis, poor bone quality due to prior surgeries or to disuse of the affected limb, or bone defects resulting from prosthesis explantation. Moreover, some data suggest that the presence of an extended trochanteric osteotomy, which can be done to remove well-fixed implants, may significantly increase the risk of a peri-spacer fracture [7, 25].

Most of the femoral fractures generally occur at the time of implant removal and are not related to the use of a cement spacer. These fractures do not require immediate treatment and are usually managed at the second stage with the use of modular revision stems and cable wires [27]. However, the surgeon should be prepared for a possible femoral fracture or fissure at the time of reimplantation. Fractures can be bridged using a long-stem revision implant, with cerclage in two cases of oblique fracture. In the series by Faschingbauer et al. [15], one out of two peri-spacer fractures was managed operatively. Differently, none of the complications required surgery before the scheduled second stage in the series by Erivan et al. [14].

Acetabular lysis or pelvic protrusion has been reported only by a few studies [2, 14–16, 18, 39, 42]. Moreover, most of them differently defined this complication. Cabrita et al. [2] observed a pelvic migration of the spacer and subsequent injury of the iliac vessels and death in one patient. Thus, they suggested not to place the spacer as a unipolar prosthesis in patients with acetabular bone weakness, particularly in obese and rheumatoid patients. In this case, they recommend the placement of a cement ball with antibiotics that fills the acetabular cavity and that articulates with the component implanted in the femoral region. Magnan et al. [18] observed that 3 out of 10 patients had a type IIA bone defect according to Paproski classification, presenting with generalized enlargement of the acetabulum and showing superomedial migration of the cup and metaphyseal femoral bone loss [18].

Konstantinos et al. [53] identified factors for increased mechanical complications risk: patient non-compliance, badly tolerated partial weight-bearing, severe acetabular bone defect, and muscular insufficiency. They found that age emerged as an important risk factor, and the appropriateness of using a spacer in elderly patients needs careful consideration. However, results from this review did not show any correlation between patients’ age and mechanical complications rate.

Unfortunately, the spacer persistence time was reported in only 28 studies and it was frequently reported as an interval rather than a mean value. This severely limits the strength of the conclusions obtainable from the analysis of this variable. Considering this limitation, no significant correlation was found between the spacer persistence time and the number of mechanical complications. Nonetheless, a strong trend has emerged as complications increase as a function of persistence time, particularly for manually molded spacers. These data can be intuitively motivated and reinforce the principle of favoring the use of the spacer in cases for which there is a real expected benefit and for which a limited persistence over time is conceivable.

A major limitation must be acknowledged, as this review was limited to major databases. However, cross-reference search should have limited this bias. Moreover, some data were not reported or very heterogenous in some of the series included.

Spacer placement in chronic PJI of the hip with bone and soft-tissue defects is challenging and bears a high risk of mechanical failures and progressive bone loss during the interim period. Moreover, the clinical superiority of the local antimicrobial therapy delivered by the spacer remains unclear, particularly in the presence of resistant pathogens [57]. A prolonged implantation period might actually endanger the outcome of the treatment, since subtherapeutic levels of antibiotics might be eluted from the spacer, and the antibiotic-impregnated cement itself provides an excellent environment for the development of resistant bacterial strains [58]. Unfortunately, spacer implantation period reported in the literature is very heterogeneous, thus making any analysis on the effect of spacer in situ time on mechanical complications impossible.

Current research shows that mortality after the first or second stage has previously been underestimated [20]. A non-spacer two-stage exchange is a viable option for managing chronically infected hip arthroplasties with severe bone loss or abductor deficiency. However, only one study has appeared that compared Girdlestone and spacer implantation in the two-stage protocol [2]. Nonetheless, a recent study reported encouraging results with a staged total hip arthroplasty protocol without spacer placement for destructive septic arthritis of the hip [59]. Therefore, a prospective study which directly compare hip two-stage procedures for hip PJI with or without a spacer is required.

## Conclusion

This review highlights a high rate of mechanical complications with the use of hip spacers in the two-stage revision surgery, mostly with the implantation of standardized molded spacers.

A careful patient selection for spacer implantation is advisable, with possible restricted indications in patients who are at risk of infection persistence, as well as those unfit for a second surgery. In these patients, a Girdlestone procedure at the time of prosthesis removal should be considered. Complications tend to increase as a function of the persistence time of spacers, thus favoring the use of the spacer in cases for which there is a real expected benefit and for which a limited persistence over time is conceivable.
